# A Practical Manual, Containing a Description of the General Chemical and Microscopical Characters of the Blood, and Secretions of the Human Body, as Well as of Their Components, Including Both Their Healthy and Diseased States; with the Best Methods of Separating and Estimating Their Ingredients; Also, a Succinct Account of the Various Concretions Occasionally Found in the Body, and Forming Calculi

**Published:** 1844-07

**Authors:** 


### ART. XXII.

*A Practical Manual, containing a Description of the general Chemical and Microscopical Characters of the Blood, and Secretions of the Human Body, as well as of their Components, including both their Healthy and Diseased States; with the best methods of separating and estimating their Ingredients; also, a succinct account of the various concretions occasionally found in the body and forming calculi.* By JOHN WILLIAM GRIFFITH, M.D. F.L.S. &c.—*London*, 1843. 12mo, pp. 62. With Two Plates.

THE plan and execution of this Manual appear to us to be alike excellent, so far as they have yet been carried out; but we have been grievously disappointed,—as we doubt not that some of its purchasers must have been,—in the limited performance of the promises set forth in the ample title-page. For it would scarcely be imagined from what there appears, that the little volume before us is solely devoted to the examination of the characters of the urine and its deposits in health and disease, and of those of vesical calculi; and that we must wait for future treatises, (of the appearances of which no definite promise is given,) before we can learn what we desire in regard to the blood and the other secretions. If this Manual had been announced as a description of the chemical and microscopical characters of the urine, &c., and as the first of a series of treatises intended to give a similar account of the other animal fluids, we should have received it with a hearty welcome. But we feel it our duty to put our readers in possession of its real scope, that they may not be disappointed in finding only a description of urine, when they were seeking for an account of blood, bile, or pus.

We have no hesitation, however, in strongly recommending this Manual as "very good as far as it goes." It will be found particularly useful to those who are working out the physiology and pathology of the urinary excretion; a department of inquiry, which, we venture to say, has not yet been pursued with that combination of previous acquaintance with the phenomena of health and disease, that philosophic acumen, and that practical skill, which the subject eminently requires. We believe that we are only now beginning to understand the true importance of the excreting processes, as *exponents* (so to speak) of the previous vital operations; and nothing but a full and complete examination of their products, and of the blood whence they are derived, can elucidate the numerous questions at issue. In this examination, so far as the urine is concerned, Dr. Griffith's Manual will prove a valuable guide; and we can therefore conscientiously give it our strong recommendation.

